# Maintaining nitrogen balance under salt stress through enhanced nodule function and antioxidative defense in chickpea

**DOI:** 10.1038/s41598-025-19585-4

**Published:** 2025-10-13

**Authors:** Gurpreet Kaur, Satish Kumar Sanwal, Nirmala Sehrawat, Ashwani Kumar, Naresh Kumar, Anita Mann, Hemant Dasila, Damini Maithani, Sumit Jangra, Kahkashan Perveen, Alanoud T. Alfagham

**Affiliations:** 1 ICAR-Central Soil Salinity Research Institute, Karnal, Haryana India; 2 Maharishi Markandeshwar (Deemed to be University), Mullana, Ambala, Haryana India; 3 Department of Chemistry and Biochemistry, Eternal University, Baru Sahib, Himachal Pradesh India; 4 Department of Microbiology, Eternal University, Baru Sahib, Himachal Pradesh India; 5 Department of Microbiology, Lovely Professional University, Phagwara, India; 6 UF/IFAS Tropical Research and Education Center Homestead, University of Florida, Florida, 33031 USA; 7 Department of Botany & Microbiology, King Saud University, P.O. Box 800, Riyadh, 11495 Saudi Arabia

**Keywords:** Chickpea, Nodules, Nitrogen, Salinity, Antioxidative enzymes, Biochemistry, Plant sciences

## Abstract

Salinity is a major environmental stress that adversely affects plant growth, nodulation and nitrogen (N) metabolism. This study investigated the physiological and biochemical responses of chickpea (*Cicer arietinum* L.) genotypes under saline conditions, with a focus on nodule performance, antioxidant enzyme activity and N-fixation efficiency. Ten genotypes, including the salt-tolerant CSG 8962, were evaluated for water relations, ion homeostasis, oxidative stress markers and nitrogen assimilation parameters. Tolerant genotypes maintained higher water potential (Ψw), osmotic potential (Ψs) and relative water content (RWC) in nodules compared to sensitive ones. Salinity-induced oxidative stress, but increased activities of antioxidative enzymes—such as superoxide dismutase, catalase, ascorbate peroxidase, monodehydroascorbate reductase, dehydroascorbate reductase, glutathione reductase, peroxidase and glutathione-S-transferase—contributed to scavenging reactive oxygen species in tolerant genotypes, resulting in lower hydrogen peroxide and malondialdehyde levels and improved membrane stability. Most genotypes (except DCP 92 -3, BG 256 and ICC 4463) maintained the Na^+^/K^+^ ratio below one, a threshold essential for cellular viability. Leghaemoglobin content decreased significantly under salinity, especially in sensitive genotypes. Although nitrate reductase (NR) activity increased with salinity, correlation analysis revealed that N accumulation in shoots was primarily due to biological nitrogen fixation rather than NR-mediated assimilation. These findings suggest that enhanced antioxidant defenses and maintenance of functional nodules are crucial for sustaining N balance in chickpeas under saline conditions.

**Supplementary Information:**

The online version contains supplementary material available at 10.1038/s41598-025-19585-4.

## Introduction

Salinity, characterized by the excessive accumulation of soluble salts in soil or water, is a major abiotic stressor that adversely affects the growth, development and productivity of agriculturally important crops worldwide^1,2^. The primary consequences of salinity include osmotic stress, which induces water deficit and ionic toxicity, particularly due to the accumulation of sodium (Na^+^) and chloride (Cl⁻) ions^3^. These conditions disrupt cellular homeostasis, leading to secondary effects such as nutritional imbalances, oxidative stress, hormonal dysregulation, inhibition of metabolic processes and premature senescence^4^. Collectively, these physiological and biochemical disturbances result in reduced biomass accumulation, impaired reproductive development and ultimately, diminished crop yields^5^.

Chickpea (*Cicer arietinum* L.), a member of the Fabaceae family, is a vital leguminous crop cultivated in tropical, subtropical and temperate regions. It serves as a critical source of plant-based protein and is enriched with essential biomolecules that confer numerous health benefits^6^. Chickpea exhibits a unique capacity for symbiotic nitrogen (N) fixation through its association with *Rhizobium* bacteria, reducing reliance on synthetic N fertilizers^7^. This symbiotic relationship can fulfill up to 85% of the plant’s N requirements, making chickpea an environmentally sustainable crop^7^. However, the excessive use of synthetic N fertilizers in modern agriculture has led to soil acidification, groundwater contamination and increased emissions of nitrous oxide, a potent greenhouse gas, thereby disrupting the global N cycle^5^.

The process of symbiotic N fixation in chickpea involves a complex interplay between the host plant and rhizobia, encompassing root hair infection, nodule formation and the enzymatic conversion of atmospheric N_2_ into ammonia via nitrogenase^8^. Salinity stress severely impairs these processes by damaging root hairs, reducing rhizobial survival and disrupting nodule development and function^9,10^. Recent studies have highlighted that salinity-induced oxidative stress further exacerbates these effects by degrading leghemoglobin, a critical protein for maintaining anaerobic conditions in nodules, thereby inhibiting nitrogenase activity^10^. Additionally, salinity disrupts ion homeostasis, particularly the Na⁺/K⁺ ratio, which is vital for cellular integrity and metabolic functions^11,12^.

Plants have evolved multifaceted mechanisms to mitigate salinity stress, including osmotic adjustment, ion exclusion and the upregulation of antioxidative enzymes such as superoxide dismutase (SOD), catalase (CAT) and ascorbate peroxidase (APX)^13^. While extensive research has focused on salinity tolerance mechanisms in leaves and roots, the responses of nodules—the sites of N fixation—remain less explored. Understanding these responses is crucial, as nodules are highly sensitive to salinity and their dysfunction directly compromises the plant’s N supply^9^.

The present study aims to address this gap by investigating the physiological, biochemical and molecular responses of chickpea nodules to salinity stress. Specifically, the objectives are (1) to evaluate the impact of salinity on nodule growth, water relations and antioxidative enzyme activities, (2) to assess the effects of salinity on N-related parameters, including leghemoglobin content, nitrate reductase (NR) activity and total N accumulation and (3) to elucidate the relationship between nodule health and N content in aboveground plant parts under saline conditions. By integrating recent advancements in plant stress biology and omics technologies (e.g., transcriptomics and proteomics), this study provides novel insights into the mechanisms underpinning salinity tolerance in chickpea nodules. The findings will contribute to the development of salt-tolerant chickpea genotypes, ensuring sustainable productivity in salinity-affected agroecosystems.

## Materials and methods

### Plant material

Seed material of nine chickpea genotypes—BG 1103, JG 16, S7, ICCV 10, DCP 92-3, KWR 108, BG 256, K 850 and ICC 4463—along with the salt-tolerant check variety CSG 8962 (Karnal Chana 1), was procured from the ICAR–Central Soil Salinity Research Institute (ICAR-CSSRI), Karnal, Haryana, India. The pedigrees of these genotypes have been detailed in our previous publication^14^. The collection and use of plant materials adhered to all relevant institutional, national and international guidelines and regulations.

### Seed pre-treatment

The seeds were treated with a *Rhizobium* culture (manufactured by Habitat Krishi, Hisar), obtained from the Kisan Sewa Kendra, Chaudhary Charan Singh Haryana Agricultural University, Haryana, India, at a concentration of 10⁸ CFU/mL. Following the manufacturer’s protocol, a solution was prepared by dissolving 1 g of jaggery in 25 mL of double-distilled water, to which 1 mL of the *Rhizobium* broth culture was added. Subsequently, 1 mL of the resulting working solution was applied to 100 g of seeds placed in Petri plates. After thorough mixing to ensure uniform coating, the seeds were air-dried at room temperature and sown within 3 h of treatment.

### Experimental set up

The experiment was carried out at the ICAR–Central Soil Salinity Research Institute, Karnal, Haryana, India. Porcelain pots (20 kg capacity) were filled with acid-washed sand and arranged in a randomized complete block design (RCBD) with three replications. Two salinity treatments were applied: medium salinity (EC_iw_ 6 dS m^− 1^) and high salinity (EC_iw_ 9 dS m^− 1^), in addition to a control treatment using the best available non-saline water. The composition of both saline and non-saline irrigation waters was previously reported in our earlier publication^15^. Naturally saline water was sourced from Nain, Panipat, Haryana and diluted as required to achieve the desired electrical conductivity levels. Prior to sowing, pots were saturated with 3.12 L of the respective irrigation water, calculated based on pot volume and bulk density. Thereafter, irrigation was applied as needed using the designated saline or control water, each supplemented with a nitrogen-free nutrient solution^16^.

### Sampling

Sampling was performed in three biological replicates. Plants were carefully uprooted at the flowering stage and the nodules were detached and thoroughly washed to remove any adhering sand particles.

### Nodule number and weight

The total number of nodules per plant was recorded. Subsequently, the nodules were oven-dried at 65 °C for 72 h and their dry weight was measured.

### Water relations

#### Relative water content (RWC)

For RWC analysis, freshly collected nodule samples were gently blotted to remove any surface moisture and the fresh weight (FW) was recorded. The turgid weight (TW) was determined after immersing the samples in double-distilled water for 4 h at 25 °C. The dry weight (DW) was measured after oven-drying the samples at 65 °C for 72 h. RWC (%) was calculated using the following formula^17^:

$$RWC~\left( \% \right)=~\frac{{\left( {FW - DW} \right)}}{{\left( {TW - DW} \right)}}~ \times 100$$

#### Water potential (Ψ_w_)

Water potential was measured using freshly harvested nodules sealed in a WP4C Dewpoint Potentiometer (METER Group, Inc., USA). The values were recorded in negative megapascals (− MPa).

#### Osmotic potential (Ψ_s_)

For osmotic potential determination, frozen nodules were crushed and the extracted cell sap was used to measure osmolality using a Vapor Pressure Osmometer (Model 5600, ELITech Group, Belgium)^18^. The osmolality (mmol/kg) was then converted to osmotic potential using the Van’t Hoff equation^19^.

$$\Psi {\text{s }}\left( {{\text{MPa}}} \right){\text{ }}={\text{ }} - {\text{c x 2}}.{\text{58 x 1}}{0^{ - {\text{3}}}}$$

### Oxidative stress indicators

An extract was prepared using 0.1% (w/v) trichloroacetic acid (TCA) for the estimation of malondialdehyde (MDA) and hydrogen peroxide (H_2_O_2_) contents^20^.

#### MDA Estimation

For MDA quantification, 1 mL of the extract was mixed with 2 mL of 0.5% thiobarbituric acid (TBA) prepared in 20% TCA. The reaction mixture was incubated at 100 °C for 30 min, then cooled to room temperature and centrifuged at 10,000 rpm for 5 min. The absorbance of the supernatant was recorded at 532 nm and 600 nm using the control as a blank. MDA content was calculated using an extinction coefficient of 155 mM^− 1^cm^− 1^.

#### H_2_O_2_ estimation

For the estimation of hydrogen peroxide, 1 mL of the extract was added to 1 mL of 10 mM potassium phosphate buffer (pH 7.0) and 2 mL of 1 M potassium iodide. The mixture was thoroughly mixed and the absorbance was measured at 390 nm using a control as blank. A standard curve was prepared using known concentrations of H_2_O_2_ to quantify the results.

#### Membrane stability index (MSI)

Membrane stability was assessed using the electrical conductivity (EC) method^21^. Freshly collected nodules (100 mg) were immersed in 20 mL of distilled water and incubated at room temperature for 2 h. Initial EC (EC_1_) was recorded. Subsequently, the samples were boiled at 100 °C for 1 h, cooled to room temperature and final EC (EC_2_) was measured. Membrane stability was calculated using the following formula:

$$MSI=\frac{{\left( {EC~after~boiling} \right)}}{{\left( {EC~before~boiling+EC~after~boiling} \right)}} \times 100$$

### Assay for enzymatic antioxidants

Fresh nodules (300 mg) were homogenized in 3 mL of 0.1 M potassium phosphate buffer (pH 7.0). The homogenate was centrifuged at 12,000 rpm for 15 min and the resulting supernatant was used for enzymatic assays.

#### Superoxide dismutase (SOD, EC 1.15.1.1)

SOD activity was estimated following the inhibition of nitro blue tetrazolium (NBT) photoreduction method^22^. Absorbance was measured at 560 nm and the activity was calculated using an extinction coefficient of 167 mM^− 1^ cm^− 1^. One enzyme unit (EU) was defined as the amount of enzyme required to inhibit the photoreduction of 1 µmol of NBT per minute^23^. The activity was expressed as EU/g fresh weight (FW).

#### Catalase (CAT, EC 1.11.1.6)

CAT activity was assayed by monitoring the decomposition of H_2_O_2_ at 240 nm for 2 min using a kinetics-based method^24^. The activity was calculated using an extinction coefficient of 0.036 mM^− 1^ cm^− 1^ and expressed as EU/g FW, where one enzyme unit corresponds to the decomposition of 1 mmol of H_2_O_2_ per mL per minute.

#### Ascorbate peroxidase (APX, EC 1.11.1.11)

APX activity was measured by monitoring the decrease in absorbance at 290 nm due to the oxidation of ascorbate^25^. The extinction coefficient used for the calculation was 2.8 mM^− 1^cm^− 1^. The activity was expressed as EU/g FW, where one enzyme unit is defined as the amount of enzyme required to oxidize 1 nmol of ascorbate per minute.

#### Peroxidase (POX, EC 1.11.1.7)

POX activity was determined by measuring the formation of tetra-guaiacol, which results in an increase in absorbance at 470 nm. An extinction coefficient of 26.6 mM^− 1^cm^− 1^ was used for the calculations. The activity was expressed as EU/g FW, where one enzyme unit represents the amount of enzyme required to oxidize 1 nmol of guaiacol per minute per mL^26^.

### Analysis of ionic composition

Well-dried nodule powder (50 mg) was digested with 80% nitric acid for the estimation of sodium (Na⁺) and potassium (K⁺) ion contents. The flame photometer (Model PFP7, Jenway, Bibby Scientific, UK) was calibrated using standard solutions of sodium chloride (NaCl) and potassium chloride (KCl) for Na⁺ and K⁺, respectively. Ion concentrations were calculated using standard protocols^14^ and expressed as percentages.

### Leghemoglobin content

Leghemoglobin content was estimated from nodules (0.5 g) homogenized in 10 mL of ice-cold 0.1 M potassium phosphate buffer (pH 7.5) using a chilled mortar and pestle, as described by Hartree et al.^27^. The homogenate was centrifuged at 5,000 rpm for 30 min at 4 °C. An aliquot of the supernatant (2 mL) was mixed with 2 mL of 0.2 M sodium hydroxide (NaOH) and incubated at room temperature for 1 h. Subsequently, 1 mL of pyridine and 50 mg of sodium dithionite (Na₂S₂O₄) were added and the mixture was incubated again at room temperature for 1 h. After incubation, the mixture was centrifuged at 5,000 rpm for 20 min at 4 °C. The absorbance of the supernatant was measured at 555 nm using an appropriate blank. Leghemoglobin content was quantified using a standard curve of hemoglobin and expressed as mg/g fresh weight (FW) of nodules.

### Nitrate reductase (NR) assay

Nitrate reductase (NR) activity was estimated using the in vivo assay method^28^. For each sample, two test tubes were prepared and labelled as T_0_ and T_30_. Fresh plant tissue (300 mg) was added to each tube, followed by 10 mL of the in vivo assay solution. The T_0_ set was immediately incubated in a boiling water bath for 5 min to terminate enzyme activity, then cooled to room temperature. Both T_0_ and T_30_ tubes were then incubated at 30 °C for 30 min to allow enzymatic conversion of nitrate to nitrite. Following this, both sets were again incubated in a boiling water bath for 5 min to stop the reaction and then cooled to room temperature. To develop color, 5 mL of 1% sulphanilamide (prepared in 3 N HCl) and 5 mL of 0.02% N-(1-naphthyl) ethylenediamine dihydrochloride (NEDA) were added to each tube. The contents were mixed thoroughly and incubated at room temperature for 5 min. Absorbance was measured at 540 nm using a control as the blank. The amount of nitrite formed was quantified using a standard curve prepared with potassium nitrite and nitrate reductase activity was expressed based on the rate of nitrate reduction to nitrite.

### Total N content

Total nitrogen (N) content was estimated using the Kjeldahl method, which is based on the principle that strong acid digestion releases nitrogen present in the sample, which is then quantified through a suitable titration technique^29^. For digestion, 250 mg of well-dried, powdered plant material was transferred into a 250 mL long-neck digestion tube. To each tube, 10 mL of concentrated sulfuric acid (H_2_SO_4_) and 500 mg of a catalytic accelerator were added. The tubes were placed on a preheated digestion block maintained at 400 °C and digested for 3.5–4 h, or until a clear, transparent residue remained. For distillation, the digestion tubes were connected to a Kjeldahl distillation unit. The automated system was programmed to sequentially add 10 mL of double-distilled water, 50 mL of an alkali reagent and 20 mL of a boric acid indicator solution. The sample was distilled for 7 min and the distillate was collected for titration. For titration, the collected distillate was titrated against 0.1 N sulfuric acid (H₂SO₄) and the volume consumed was recorded. The total nitrogen content in the sample was calculated using standard formulae and expressed as a percentage (%).

### Assessment of gene expression in response to salinity

Total RNA was extracted following the manufacturer’s instructions provided with the TRIzol reagent (HiMedia). The concentration and purity of the extracted RNA were assessed using a Nanodrop spectrophotometer (DeNovix^®^ DS-11+). To eliminate genomic DNA contamination, the RNA samples were treated with DNase I (Thermo Scientific). Following DNase I treatment, the RNA concentration and purity were re-evaluated using the same spectrophotometer. First-strand complementary DNA (cDNA) synthesis was performed using the iScript™ cDNA Synthesis Kit (Bio-Rad), following the manufacturer’s protocol.

The transcript levels of key antioxidant genes (*SOD*,* CAT*,* APX*,* MDHAR*,* DHAR*,* GR*,* POX* and *GST*), along with nitrate reductase (*NR*) and leghemoglobin (*LEG*), were analyzed. Primer sequences for GST, LEG and NR are provided in Table 1, while those for the remaining genes are available in our previously published study^14^. Quantitative real-time PCR was performed using the SsoAdvanced™ Universal SYBR^®^ Green Supermix (Bio-Rad) in three biological replicates to assess gene expression levels. The relative fold change in transcript abundance was calculated using the 2^−ΔΔCt^ method^30^.

**Table 1:** Primer sequences used for quantitative expression in Chickpea nodules.

Gene	Primer sequence	Amplicon size (bp)	Annealing temperature	Accession number
*GST*	FP-*GTT GTG GTC AGA AGT GGA GAG*	124	54	XM_004495920.3
	RP-*GGC TTC ATT CTC TTC CAC AAC*			
*LEG*	FP-*CTT CTA GCA AAA GGT ACA GTA G*	153	52	XM_004490852.3
	RP-*GAG TTC TTC GCT CCA TGT G*			
*NR*	FP-*CCT CAG TGT TAG ATC CTC G*	111	52	XM_004500543.2
	RP-*GTA GTG GTG GCT CTG AG*			

### Correlation analysis

Correlation analysis between various physiological and morphological traits of nodules and the total nitrogen content in leaves was performed using PAST software (Version 4.03).

### Statistical analysis

All measured and computed data (in triplicates) were subjected to statistical analysis using SAS software (Version 9.3, SAS Institute Inc., USA). Two-way factorial analysis of variance (ANOVA) was performed to assess the significance of treatment effects and interactions. Mean comparisons were conducted using Tukey’s Honest Significant Difference (HSD) test at a 5% probability level (*p ≤ 0.05*).

## Results

Significant variability for various studied traits among genotypes under different treatments was noticed in the present study.

### Nodule formation and growth

Salinity stress exerted an inhibitory effect on nodule formation and growth as it reduced with salinity (Table 2). Among the studied genotypes, K 850 was found to be highly nodulating as maximum number of nodules were found in this genotype under all the three conditions i.e., control, EC_iw_ 6 dS m^− 1^ and EC_iw_ 9 dS m^− 1^. Nodule formation was highly affected in ICC 4463 as minimum number of nodules were attached to the roots of this genotype under salinity (EC_iw_ 6 dS m^− 1^ and EC_iw_ 9 dS m^− 1^). Both fresh and dry weights of nodules were affected by saline irrigation. Under control conditions genotypes BG 256, S7 and K 850 showed maximum fresh weight of 6.03, 6.22 and 6.57 (g) respectively. Genotypes ICCV 10 and KWR 108 showed minimum reduction in fresh weight at EC_iw_ 6 dS m^− 1^ (23.53% and 24.63%) and EC_iw_ 9 dS m^− 1^ (46.82% and 47.62%) respectively. Similarly, dry weight of the nodules also reduced with salinity and minimum reduction was observed in the genotype ICCV 10 (23.27%) and maximum in genotype ICC 4463 (52.10%) at EC_iw_ 6 dS m^− 1^, while higher saline water irrigation (EC_iw_ 9 dS m^− 1^) caused minimum reduction in the genotype KWR 108 (47.49%) and maximum reduction in genotype ICC 4463 (86.23%) *w.r.t.* control.

**Table 2:** Effect of salinity on number and weight of nodules.

Source of variation	DF	Mean square								
		Nodule number			Nodule fresh weight (g)			Nodule dry weight (g)		
Replication	2	0.1778			0.0027			0.0005		
Treatment (T)	2	3812.15**			102.03**			1.05		
Genotype (G)	9	236.87**			3.14**			0.0346		
G x T	18	40.61**			0.7019**			0.0053		

Treatment/Traits		C	${\text{EC}}_{{{\text{iw}}}}$ 6 dSm^-1^	${\text{EC}}_{{{\text{iw}}}}$ 9 dSm^-1^	C	${\text{EC}}_{{{\text{iw}}}}$ 6 dSm^-1^	${\text{EC}}_{{{\text{iw}}}}$ 9 dSm^-1^	C	${\text{EC}}_{{{\text{iw}}}}$ 6 dSm^-1^	${\text{EC}}_{{{\text{iw}}}}$ 6 dSm^-1^
CSG 8962		46.34^ab^	34.67^b^	26.34^abcd^	5.6b^cde^	4.13^abc^	2.82^a^	0.58^bcd^	0.42^bcd^	0.26^b^
BG 1103		47.34^ab^	30.67^b^	19.34^e^	5.89^bcd^	3.90^bcd^	1.13^bc^	0.61^abc^	0.38^cde^	0.14^c^
S7		47.67^ab^	35.34^b^	27.34^abc^	6.22^ab^	4.58^a^	3.13^a^	0.63^ab^	0.48^ab^	0.30^ab^
DCP 92-3		47.34^ab^	34.00^b^	20.34^de^	5.83^bcd^	3.78^cd^	1.50^b^	0.59^bcd^	0.38^cde^	0.17^c^
ICCV 10		38.34^c^	32.00^b^	22.00^bcde^	5.40^de^	4.12^abc^	2.87^a^	0.53^de^	0.41^cde^	0.27^ab^
KWR 108		43.34^bc^	35.34^b^	27.67^ab^	5.67^bcde^	4.27^abc^	2.97^a^	0.59^bc^	0.44^abc^	0.32^ab^
BG 256		44.34^bc^	31.67^b^	23.34^abcde^	6.03^abc^	3.45^d^	1.23^bc^	0.60^bc^	0.35^e^	0.14^cd^
K 850		51.67^a^	45.00^a^	29.34^a^	6.57^a^	4.48^ab^	3.24^a^	0.67^a^	0.50^a^	0.33^a^
JG 16		38.67^c^	31.34^b^	21.34^cde^	5.05^e^	3.49^d^	1.23^bc^	0.52^e^	0.36d^e^	0.12^cd^
ICC 4463		42.67^bc^	18.67^c^	5.34^f^	5.43^cde^	2.72^e^	0.68^c^	0.56^cde^	0.27^f^	0.07^d^
HSD @ 5%		6.0445			0.6235			0.0636		

Values represent the mean from three independent experiments; Superscripted alphabets denote the significance level at *P* < 0.05.

### Water relations in nodules

**Fig. 1:**
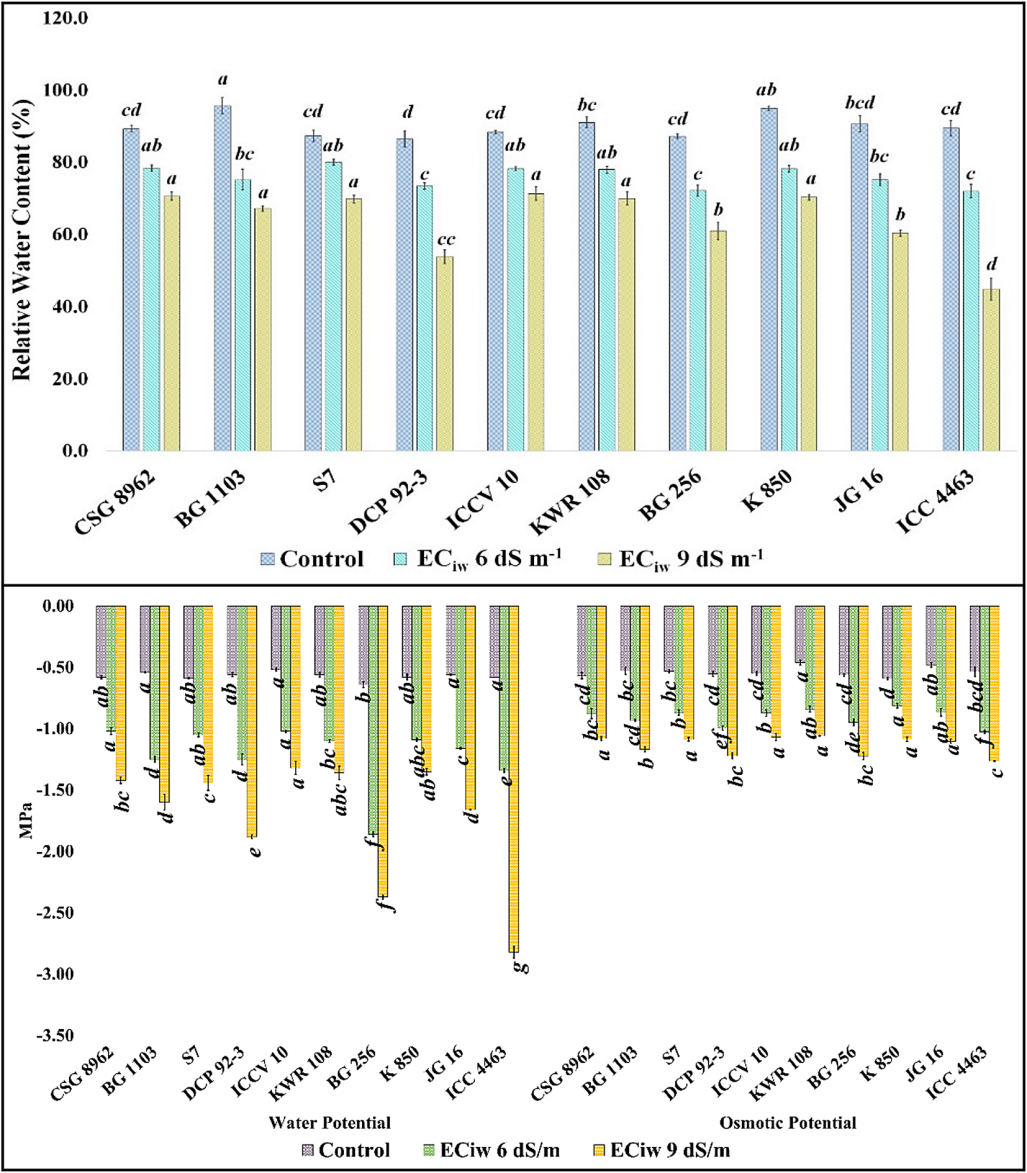
Effect of saline irrigation on relative water content, water potential (ψ_w_) and osmotic potential (ψ_s_) (Superscripted alphabets denote the significance level at P < 0.05).

Osmotic phase of salinity creates drought like conditions and thus traits related to water is important to consider. Reduction in RWC with salinity is a common phenomenon and the effect was also observed in chickpea nodules (Fig. 1). A mean genotypic reduction of 15.44% and 29.03% was observed at EC_iw_ 6 dS m^− 1^ and 9 dS m^− 1^ respectively w.r.t control. Maximum reduction was shown by the nodules of the genotype BG 1103 (21.45%) at EC_iw_ 6 dS m^− 1^ whereas at EC_iw_ 9 dS m^− 1^ maximum reduction was observed in the genotype ICC 4463 (49.85%) (Fig. 1). ψ_w_ and ψ_s_ are the other two important traits to be considered under salinity. Both ψ_w_ and ψ_s_ became more negative with salinity (Fig. 1). None of the studied genotype showed less than − 0.7 ψ_w_ at control environment but at EC_iw_ 6 dS m^− 1^ it became more negative and genotypes BG 1103, DCP 92 − 3, BG 256 and ICC 4463 showed less than − 1.20 value for ψ_w_. High salinity (EC_iw_ 9 dS m^− 1^), further reduced the ψ_w_ and genotypes BG 256 and ICC 4463 showed more negative values than − 2. Similarly, ψ_s_ reduced with salinity and at EC_iw_ 6 dS m^− 1^ only genotype ICC 4463 showed more negative value than − 1 and at EC_iw_ 9 dS m^− 1^, all the genotypes showed more negative value than − 1 but genotypes ICCV 10 and KWR 108 showed less negative value for ψ_s_ than the salt tolerant check CSG 8962.

### Oxidative stress indicators

Salinity created oxidative stress in chickpea nodules as the content of H_2_O_2_ increased with salinity which is one of the reactive oxygen species (ROS). Moderate saline level (EC_iw_ 6 dS m^− 1^), raised the H_2_O_2_ content by more than thrice in the genotypes DCP 92 − 3 and ICC 4463 w.r.t control whereas at high salinity level (EC_iw_ 9 dS m^− 1^) the values were 4 times higher than the control in these genotypes (Table 3). Excessively generated ROS damages the lipids present in the membranes by peroxidation reaction. MDA is the product of this peroxidation reaction and its content increased with rise in the concentration of the salts in the irrigation water (Table 3). At EC_iw_ 6 dS m^− 1^_,_ minimum MDA content was observed in the salt tolerant check CSG 8962 (4.33 nmol/g FW) and at EC_iw_ 9 dS m^− 1^, genotypes S7, ICCV 10 and KWR 108 showed less MDA content than CSG 8962. Membrane stability is another factor to know the degree of damage caused by ROS. Mean genotypic membrane stability under control conditions was 73.94 which reduced to 63.75 and 57.20 at EC_iw_ 6 and 9 dS m^− 1^ respectively.

**Table 3:** Effect of salinity on H_2_O_2_, MDA content and membrane stability.

Source of variation	DF	Mean square								
		H_2_O_2_ (µmoles/g FW)			MDA (nmol/g FW)			Membrane stability (%)		
Replication	2	0.1614			0.0792			4.7986		
Treatment (T)	2	3609.18**			191.99**			2133.57**		
Genotype (G)	9	123.04**			5.17**			71.05**		
G x T	18	34.55**			1.56**			24.00**		

Treatment/Traits		Control	${\text{EC}}_{{{\text{iw}}}}$ 6 dSm^-1^	${\text{EC}}_{{{\text{iw}}}}$ 9 dSm^-1^	Control	${\text{EC}}_{{{\text{iw}}}}$ 6 dSm^-1^	${\text{EC}}_{{{\text{iw}}}}$ 9 dSm^-1^	Control	${\text{EC}}_{{{\text{iw}}}}$ 6 dSm^-1^	${\text{EC}}_{{{\text{iw}}}}$ 6 dSm^-1^
CSG 8962		9.31^cde^	20.59^fg^	28.58^e^	2.23^c^	4.34^e^	7.52^de^	75.71^ab^	65.15^ab^	61.40^a^
BG 1103		11.27^ab^	28.92^c^	35.39^c^	2.33^c^	5.79^bc^	8.18^cd^	72.48^bc^	59.33^d^	51.79^d^
S7		8.33^e^	19.12^g^	25.49^g^	3.44^a^	4.75^de^	6.37^f^	72.30^bc^	68.28^a^	60.86^ab^
DCP 92-3		9.31^cde^	32.35^b^	37.30^b^	2.45b^c^	6.86^a^	8.87^b^	71.61^bc^	59.99^cd^	53.49^cd^
ICCV 10		10.29^bcd^	22.06^ef^	27.30^ef^	3.04^ab^	4.50^e^	6.95^ef^	72.56^bc^	67.95^a^	60.23^ab^
KWR 108		12.75^a^	21.57^f^	26.08^fg^	2.52^bc^	4.58^e^	6.63^f^	77.98^a^	64.40^abc^	62.012^a^
BG 256		11.76^ab^	32.35^b^	35.49^c^	3.24^a^	6.35^ab^	9.76^a^	75.24^abc^	56.80^d^	52.84^cd^
K 850		10.78^bc^	23.53^e^	25.68^fg^	3.28^a^	4.42^e^	7.35^e^	71.03^c^	68.66^a^	59.29^ab^
JG 16		8.82^de^	25.98^d^	30.53^d^	3.37^a^	5.41^cd^	8.38^bc^	77.67^a^	65.72^ab^	56.45^bc^
ICC 4463		9.31^cde^	35.29^a^	40.15^a^	3.43^a^	6.65^a^	9.91^a^	72.79^bc^	61.22^bcd^	53.62^cd^
HSD @ 5%		1.7958			0.6634			4.5864		

Values represent the mean from three independent experiments; Superscripted alphabets denote the significance level at *P* < 0.05.

### Antioxidative enzymes

**Fig. 2:**
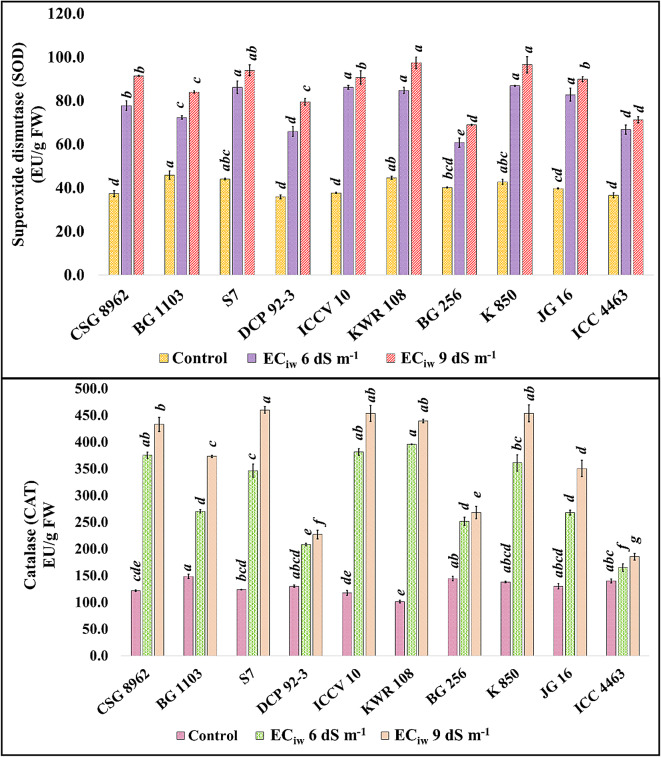
Effect of salinity on SOD and CAT activities in chickpea nodules (Superscripted alphabets denote the significance level at P<0.05).

Enhanced activity of antioxidative enzymes (SOD, CAT, APX and POX) in chickpea nodules was observed with increasing salt concentration in irrigation water. Mean genotypic SOD activity at control condition was 40.5 which increased to 77 and 86.4 at EC_iw_ 6 and 9 dS m^− 1^, respectively. Genotype BG 256 showed minimum and ICCV 10 showed maximum increment in the SOD activity at both the salinity levels with respect to control (Fig. 2). CAT activity enhanced with increasing salinity and more than 3 times enhancement in the CAT activity was noticed in the genotypes KWR 108 (3.92), ICCV 10 (3.25) and CSG 8962 (3.08) at EC_iw_ 6 dS m^− 1^ with respect to control. CAT activity further enhanced at EC_iw_ 9 dS m^− 1^ and minimum activity was observed in the genotype ICC 4463 whereas genotype KWR 108 showed maximum CAT activity (Fig. 2).

**Fig. 3:**
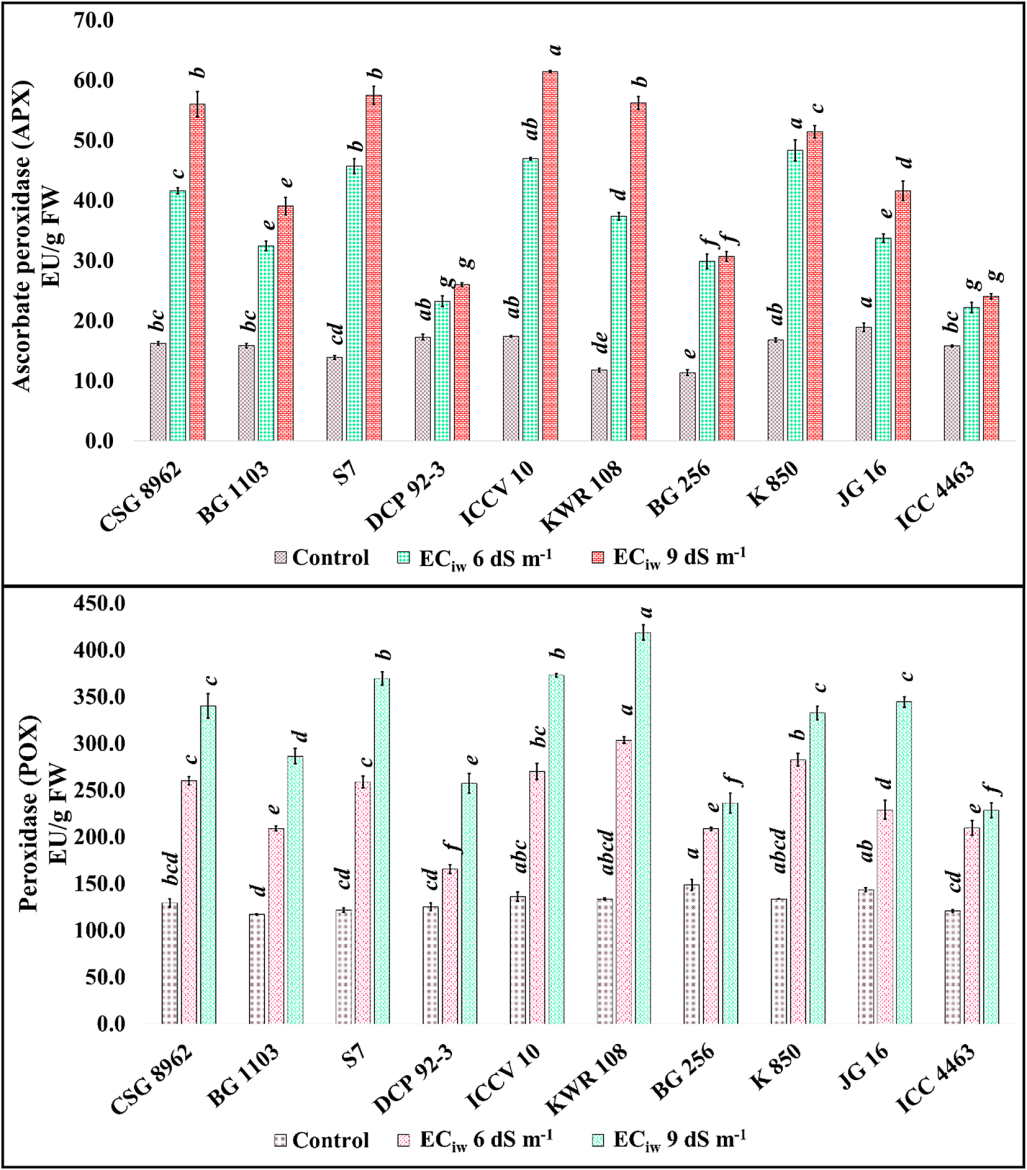
Effect of salinity on APX and POX activities in chickpea nodules *(Superscripted alphabets denote the significance level at P<0.05)*.

APX activity increased threefold in the nodules of the genotypes KWR 108 (3.17) and S7 (3.29) at EC_iw_ 6 dS m^− 1^ w.r.t control. At EC_iw_ 9 dS m^− 1^, the activity raised by 4 times in the nodules of these genotypes w.r.t control (Fig. 3). Nodules of the genotype DCP 92 − 3 showed minimum enhancement among all the studied genotypes at both the salinity levels w.r.t control. POX activity also increased with salinity in the nodules of all the genotypes (Fig. 3). At EC_iw_ 6 dS m^− 1^, minimum POX activity was observed in the genotype DCP 92 − 3 with 165.6 EU/ g FW whereas genotype KWR 108 showed maximum activity with 303.5 EU/g FW. At EC_iw_ 9 dS m^− 1^, minimum activity was observed in the genotype ICC 4463 with 228.3 EU/g FW and maximum activity was observed in the nodules of KWR 108 with 418.7 EU/g FW.

### Na^+^/K^+^ ratio

Increasing concentration of salts in irrigation water disrupted the Na^+^/K^+^ ratio in the nodules as shown in Table 4. Drastic change was observed at higher salinity level (EC_iw_ 9 dS m^− 1^), especially in the genotypes DCP 92 − 3, BG 256 and ICC 4463. Several important biological processes require K + ions but, high Na + ion content was measured in the nodules of the genotypes DCP 92 − 3, BG-256 and ICC 4463 at higher salinity level (ECiw 9 dS m-1).

**Table 4:** Effect of saline water irrigation on total nitrogen content.

Source of variation	DF	Mean square								
		Na^+^/K^+^			NR (µmol/gm FW/ hr)			Leaves total *N* content (%)		
Replication	2	0.0003			0.0157			0.0030		
Treatment (T)	2	9.3915			139.4970			11.1796		
Genotype (G)	9	1.3045			3.2002			2.2256		
G x T	18	0.8996			1.8752			0.5841		

Treatment/Traits		Control	${\text{EC}}_{{{\text{iw}}}}$ 6 dSm^-1^	${\text{EC}}_{{{\text{iw}}}}$ 9 dSm^-1^	Control	${\text{EC}}_{{{\text{iw}}}}$ 6 dSm^-1^	${\text{EC}}_{{{\text{iw}}}}$ 9 dSm^-1^	Control	${\text{EC}}_{{{\text{iw}}}}$ 6 dSm^-1^	${\text{EC}}_{{{\text{iw}}}}$ 6 dSm^-1^
CSG 8962		0.17^a^	0.32^def^	0.48^g^	2.06^ab^	3.50^a^	7.24^a^	3.24^a^	2.92^abc^	2.68^a^
BG 1103		0.16^a^	0.39^de^	0.92^d^	1.78^b^	2.56^c^	6.59^bc^	3.18^ab^	2.36^d^	1.33^d^
S7		0.19^a^	0.27^f^	0.64^ef^	1.90^ab^	3.26^ab^	7.08^a^	3.20^ab^	2.98^a^	2.63^ab^
DCP 92-3		0.16^a^	0.53^b^	2.42^c^	2.31^a^	2.15^c^	4.75^d^	3.16^ab^	1.26^e^	1.02^e^
ICCV 10		0.13^a^	0.30^ef^	0.55^fg^	1.86^b^	3.54^a^	7.00^ab^	2.94^c^	2.77^bc^	2.48^b^
KWR 108		0.15^a^	0.28^f^	0.49^g^	2.02^ab^	3.38^ab^	7.20^a^	3.13^abc^	2.96^ab^	2.65^ab^
BG 256		0.13^a^	0.50^bc^	2.57^b^	2.15^ab^	2.39^c^	3.91^e^	3.14^ab^	1.36^e^	1.16^de^
K 850		0.16^a^	0.33^def^	0.49^g^	1.90^ab^	3.38^ab^	7.33^a^	3.29^a^	2.99^a^	2.77^a^
JG 16		0.14^a^	0.42^cd^	0.72^e^	1.90^ab^	3.01^b^	6.34^c^	3.19^ab^	2.75^c^	1.72^c^
ICC 4463		0.15^a^	0.77^a^	2.98^a^	2.15^ab^	2.39^c^	3.79^e^	3.04^bc^	1.44^e^	1.01^e^
HSD @ 5%		0.0611			0.4417			0.1937		

Values represent the mean from three independent experiments; Superscripted alphabets denote the significance level at *P* < 0.05.

### Leghemoglobin content

**Fig. 4:**
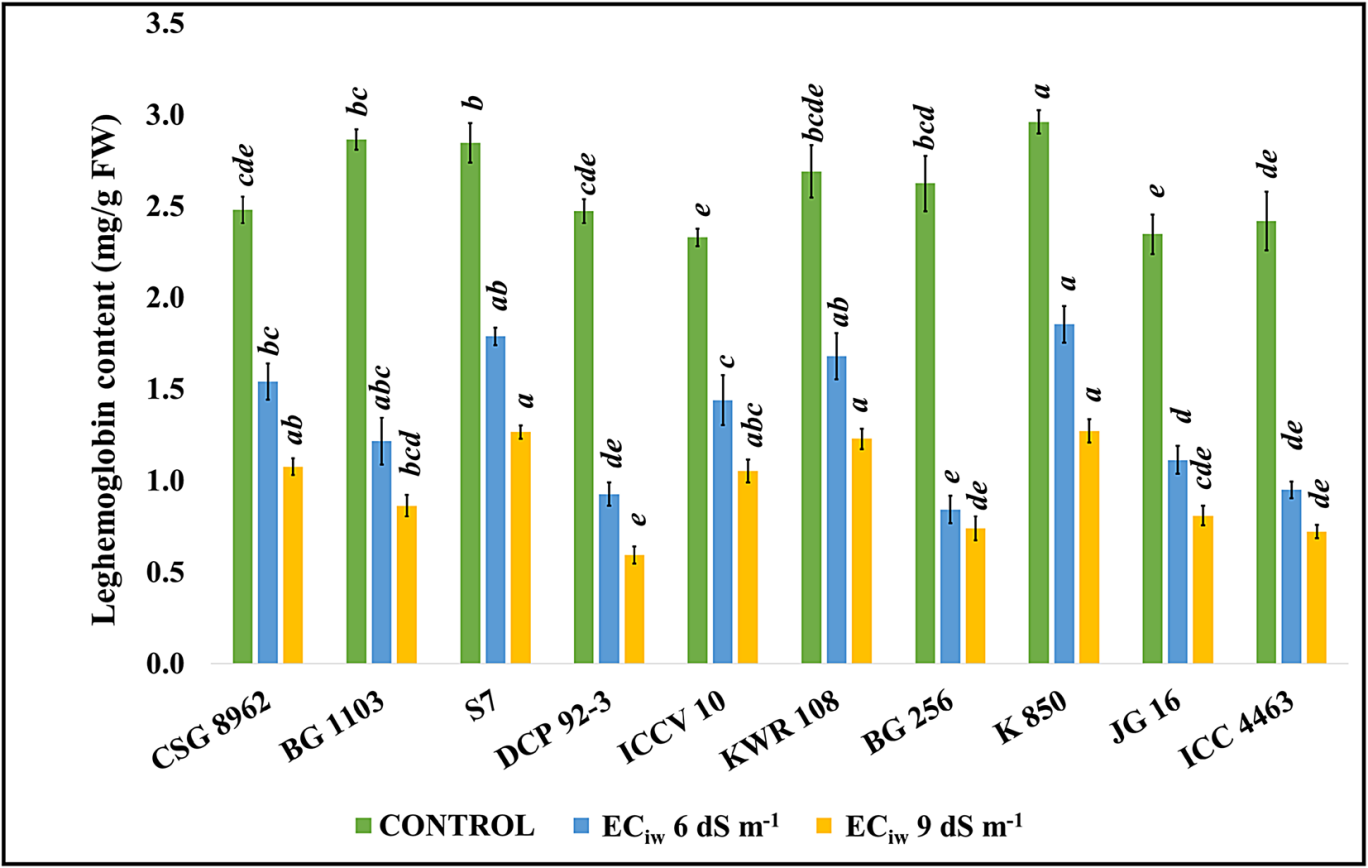
Effect of salinity on leghemoglobin content *(Superscripted alphabets denote the significance level at P<0.05)*.

The pigment imparting pink color to the nodules reduced when the plants were exposed to salinity (Fig. 4). Saline irrigation of EC_iw_ 6 dS m^− 1^ reduced the leghemoglobin content with mean genotypic reduction of 48.95%, minimum being observed in the genotype S7 (37.21%) and maximum in the genotype BG 256 (67.89%). At EC_iw_ 9 dS m^− 1^, genotypes BG 1103, S7, BG 256, JG 16, ICC 4463 showed more than 65% reduction w.r.t control.

### Nitrate reductase activity and nitrogen content

Salinity enhanced the rate of NR enzyme in chickpea nodules (Table 4). Maximum rate of NR enzyme was shown by the genotype K 850 at both the salinity levels. Salinity reduced the total nitrogen content in chickpea leaves. A mean genotypic reduction of 24.60% and 38.38% was observed at EC_iw_ 6 dS m^− 1^ and 9 dS m^− 1^ respectively with respect to control. Although total nitrogen content reduced with salinity but significant reduction was observed in the genotypes BG 1103, DCP 92 − 3, BG 256 and ICC 4463 at EC_iw_ 6 dS m^− 1^ w.r.t control. High salinity level of EC_iw_ 9 dS m^− 1^ further reduced the nitrogen content in chickpea leaves and genotypes BG 1103, DCP 92 − 3, BG 256, JG 16 and ICC 4463 showed more than 40% reduction (Table 4).

### Assessment of gene expression in response to salinity

**Fig. 5:**
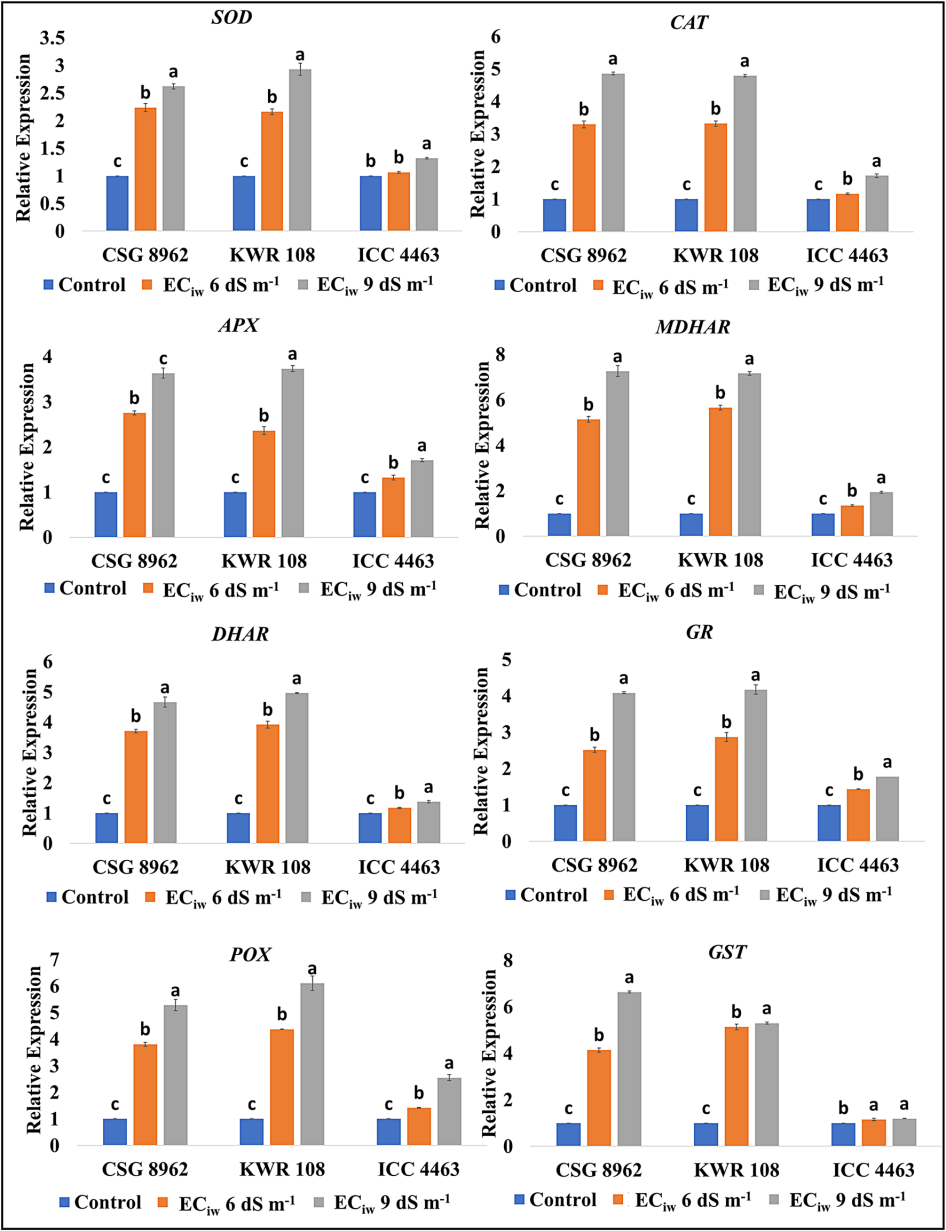
Effect of salinity on the gene expression levels of antioxidative enzymes.

Change in the transcript expression (related to the biochemical parameters) with respect to salinity was studied in the salinity contrasting genotypes (KWR 108-tolerant and ICC 4463-sensitive) along with the tolerant check for salinity (CSG 8962). Section of the salinity contrasting genotypes is based upon the present and our previous investigations^14,15,31^. Salinity induced an upregulation in the expression of the transcripts corresponding to the antioxidative enzymes in chickpea nodules (Fig. 5). Expression of Superoxide dismutase (*SOD*), Catalase (*CAT*), Ascorbate peroxidase (*APX*), Monodehydroascorbate reductase (*MDHAR*), Dehydroascorbate reductase (*DHAR*), Glutathione reductase (*GR*), Peroxidase (*POX*) and Glutathione-S-transferase (*GST*) gene was upregulated but the significant change between control and saline conditions was detected only in the tolerant chickpea cultivar (KWR 108), which was similar to check for salinity tolerance (CSG 8962) (Fig. 5).

**Fig. 6:**
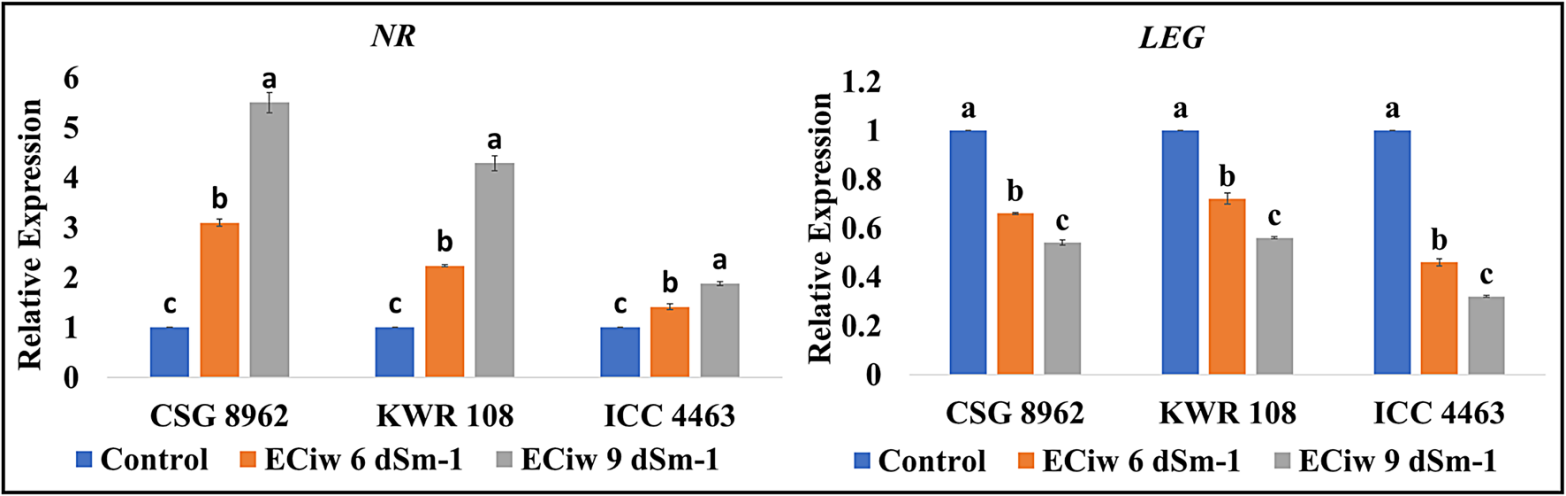
Effect of salinity on the expression levels of *NR* and *LEG* genes.

Biochemical analysis showed that the leghaemoglobin content decreased with a rise in the degree of salinity and similarly the transcript corresponding to this protein was also downregulated with salinity (Fig. 6). The LEG expression in sensitive genotype (ICC 4463) was more affected by salinity than the tolerant ones (CSG 8962 and KWR 108) (Fig. 6). The NR gene showed an upregulation in the expression with salinity. Highest upregulated expression was observed in the check for salinity tolerance (CSG 8962), followed by the salinity tolerant genotype (KWR 108) and lowest being observed in the sensitive genotype ICC 4463 (Fig. 6).

### Correlation analysis

**Fig. 7:**
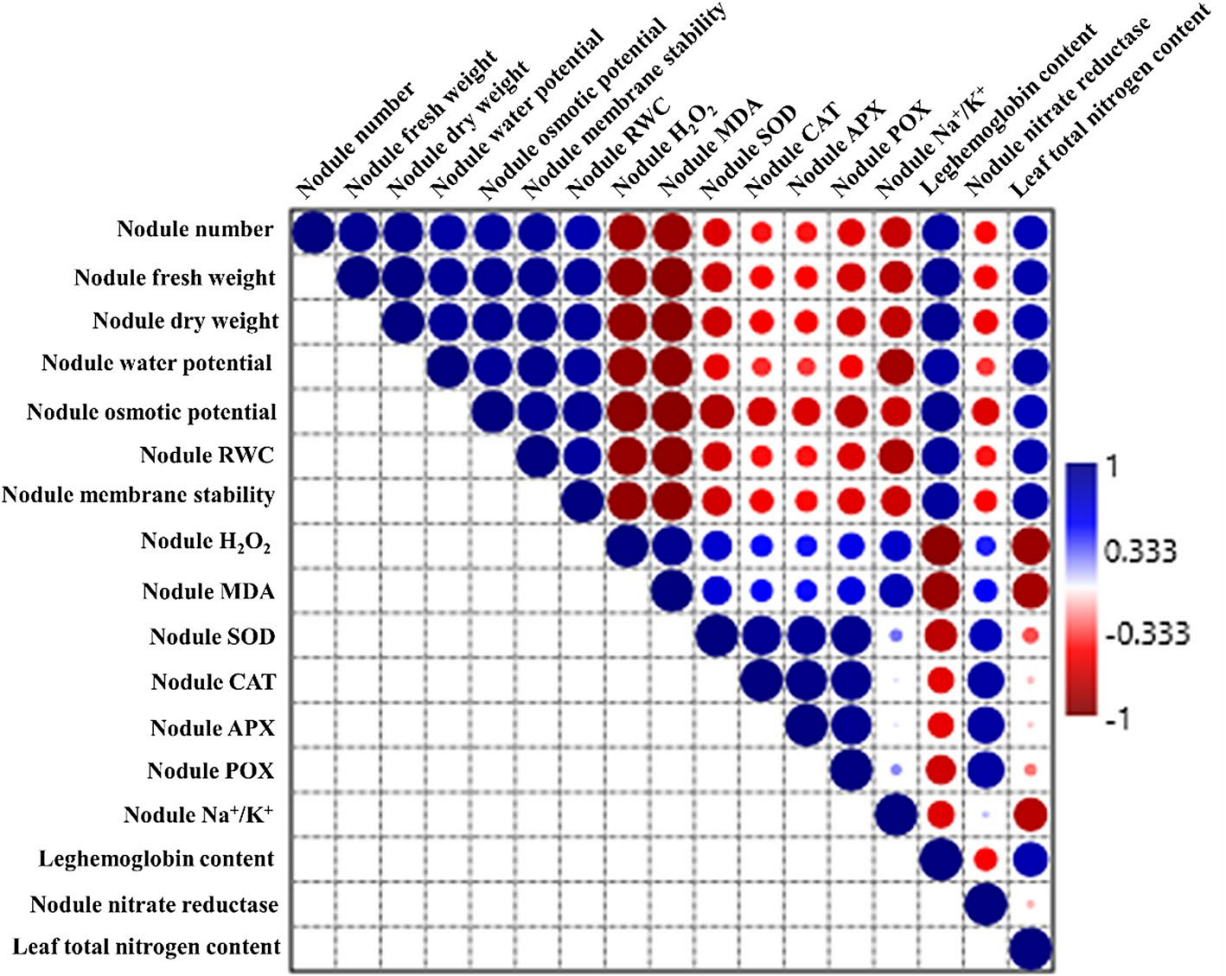
Pearson correlation coefficients among various physio-biochemical traits and total nitrogen content present in the chickpea leaves.

The relation between various physiological and morphological traits of nodules with the total nitrogen content present in leaves was studied through correlation analysis. Total nitrogen content present in leaves was found highly correlated with the nodule number, weight, RWC, water potential, osmotic potential and membrane stability (Fig. 7). Further, the leghemoglobin content was also positively correlated with the total nitrogen content. There was a strong negative correlation of H_2_O_2_ and MDA content with the leghemoglobin and total nitrogen content (Fig. 7). Surprisingly, all the antioxidative enzymes (SOD, CAT, APX and POX) and NR enzyme were negatively associated with the total nitrogen content and antioxidative enzymes and NR enzyme showed a positive association (Fig. 7).

## Discussion

Leguminous plants possess high sensitivity to salt affection and apart from other factors (germination, growth, reproduction, yield, etc.) the process of symbiotic nitrogen fixation between the *Rhizobium* bacteria and the plant is also affected. Salt affection reduced the nodule number and weight in the present investigation. Salinity stress hastens the root and bacterial symbiotic association because a halt in the root hair growth affects the infection process, reduces the survival rate of rhizobia, disrupts the nodule development and function^32^. Changes in the cytoskeleton of the root hair are responsible for the initiation of the infection process and curling of root hair entraps the bacteria but salinity severely damages the secondary roots including root hairs which automatically affects the symbiotic association between the *Rhizobium* bacteria and the plant^14,33^. Moreover, nodulation under saline conditions is quite dependent on the *Rhizobium* strain and salinity alters the cell structure and number of this bacteria which halts the process of nodule formation^34^. Nodulation in chickpea was reduced with salinity but to a lesser extent in the highly nodulating cultivar^35^. Similarly, in the present work the number of nodules decreased; however, K 850, known for its high nodulation capacity and under salinity less reduction was observed in this genotype. A report on nodule biosynthesis documented that salt stress results in the disruption of CYCLOPS-DELLA-NSP2/1-NAC181 complex, thus NAC181 independently partially activates NIN which results in the formation of fewer nodules^36^. The growth of the nodules was also affected with salinity as the reduction in the fresh and dry weight was observed. Reduction in the weight under salinity is due to the low number and volume of nodules^37^. Moreover, reduction in the photosynthetic rate, due to salinity, limits the translocation of photo assimilates to the roots which reduces the nodule growth and even formation^33^. In the present study also, the rate of photosynthesis also declined in the genotypes with less and damaged nodules (photosynthesis data not published).

The traits which deal with the plant water status are RWC, ψ_w_ and ψ_s_. Salt affection caused drastic nodular injuries and reduction in nodular RWC, ψ_w_ and ψ_s_ was observed with the hike in the degree of salinity. Similar effect of salinity on these water related traits has also been reported earlier in chickpea and investigators suggested that it was due to the reduced osmotic potential of the rhizosphere and hike in nodular ionic concentration^38^. RWC and ψ_s_ reduced with increasing salt concentration in mungbean [*Vigna radiata* (L.) Wilczek] genotypes, more being in the sensitive ones. Authors postulated that a decrease in Ψs helps the plant in pressure potential maintenance and adjustment to physiological drought like conditions generated by salinity. Moreover, accumulation of osmolytes also lower the ψ_s_^39^. There is a drastic effect of salinity on soil water potential due to which plants found it difficult to drew water from the rhizosphere and this condition leads to lowering of water and turgor potential in plants. Lowering of water potential further lowers the osmotic potential which hampers important physiological traits^4^.

Disruption of the balance between the ROS generation and scavenging leads to oxidative stress. Salinity induces a hike in ROS production, which was evident in the current investigation due to the excess content of H_2_O_2_ and MDA. Although H_2_O_2_ holds a key role in transcriptional reprogramming, plant hormone network modulation, long distance signalling, regulation of transcriptional and translational machinery, etc.^40^. At cellular concentrations, H_2_O_2_ itself is a stable biomolecule but its reaction with other molecules such as superoxide ion, nitric oxide aids in loss of protein function and gene expression, damages Fe and FeS containing proteins, oxidizes methionine residues, etc^41^. Thus, cellular H_2_O_2_ concentrations are beneficial for the stressed plants and in the present investigation it can be assumed that salinity induced increase in the H_2_O_2_ concentration to a certain level like in genotypes CSG 8962, S7, ICCV 10 and KWR 108 might have provided tolerance to the plants. In nodules, reactions catalysed by SOD and diamine oxidase enzymes are the main sources of H_2_O_2_ production^42^. Reaction between ROS and lipids in biological membranes engenders various reactive carbonyl species (RCS) including MDA and salinity induced shoot up in MDA content has been observed in this investigation also. Excessive RCS generation is tightly linked to root injury, senescence of siliques, programmed cell death and membrane stability^43^. MDA content increased and membrane stability decreased with salinity in the present study more being in the genotypes DCP 92 − 3, BG 256 and ICC 4463. Plants are equipped with an impressive powerful antioxidative system (enzymatic and non-enzymatic biomolecules) to maintain the oxidative balance. The enzymatic activity of SOD, CAT, APX and POX get accelerated with salinity in the nodules of chickpea in the present investigation. A study demonstrated that stressors lead to the generation of superoxide radicals and conversion of leghemoglobin (LB^2+^) to ferric leghemoglobin (LB^3+^) in nodules. Superoxide radicals further participates in the conversion of LB^2+^ to LB^3+^^44^. SOD, a long peptide chain metal enzyme, works to scavenge superoxide free radicals and CAT, APX, POX and other enzymes detoxify the H_2_O_2_ produced by this process and other reactions^45^. In an earlier study on salinity exposed chickpea nodules, the rate of SOD and CAT enzymes was lowered whereas the POX activity was raised with salinity^46^. A report on salinity exposed nodules of *Phaseolus vulgaris* revealed that salinity activated the SOD and POX enzymes and helped the plant to mitigate oxidative stress^47^. A salinity-based study was conducted on the nodules of chickpea genotype CSG 8962 (used as salt tolerant check in the present study) and there was induction in the specific activity of SOD, CAT, POX, APX, GR and GST^38^. Real time transcript expression of the genes encoding *SOD*, *CAT*, enzymes of ascorbate-glutathione (AsA-GSH) cycle (*APX*, *MDHAR*, *DHAR*, *GR)*, *POX* and *GST* were also studied in chickpea nodules in this investigation. Sharp upregulation with salinity was noticed in the tolerant genotypes (CSG 8962 and KWR 108) whereas a nonsignificant hike was observed in the sensitive genotype (ICC 4463). *SOD* genes have been detected in chickpea nodules and roots but the expression was more in the salinity exposed roots rather than the nodules^44^. *CAT* gene expression raised upon cadmium stress in soybean upto a particular time duration^48^. An advanced study (deepSuperSAGE) reported two APX encoding genes (*APX1* and *APX2*) in chickpea nodules and showed a direction proportion of the same with salinity^44^. Similarly, other important enzymes of the AsA-GSH pathway and POX come into play to maintain oxidative balance in nodules^42^. Escalation in the GST gene expression with salinity upswing was seen in this investigation and a transcriptomic analysis identified an upregulated transcript encoding *GST* enzyme in chickpea nodule tissue exposed to salinity. GSTs functions in glutathione mediated scavenging of peroxides where glutathione act as an electron acceptor. These enzymes also have the ability to detoxify the endogenous compounds like peroxidized lipids by conjugating glutathione to the targets and facilitates the sequestration and/or removal of such compounds^49^.

Increasing concentrations of the salts in the irrigation water disrupted the Na^+^/K^+^ ratio in the chickpea nodules more being in the sensitive genotypes. Similar results have been reported in mungbean [*Vigna radiata* (L.) Wilczek] genotypes. Authors correlated the Na^+^/K^+^ ratio with the drastic reduction in Ψs and RWC and postulated that cumulative effects of salts and reduction in Ψs and RWC caused maximum membrane injury in nodules^39^. There is competition between the Na^+^ and K^+^ ions for the entrance into the cell and Na^+^ being small in size and bearing same ionic radii enters first. The comparison of the present work and our earlier findings revealed that more Na^+^/K^+^ was more in the roots of these genotypes as compared to nodules and *HKT1* gene in the roots might be responsible for the transportation of Na^+^ ions into the roots from the nodules^14^. It is very important to maintain the ionic homeostasis in the nodules as slightly acidic conditions are required for the process of symbiotic biological N fixation^8,50^.

Leghemoglobin pigment reduced with salt affection, oxygen free condition is a necessity for the functioning of nitrogenase enzyme and this protein maintains the same in the nodules. Salt exposure reduced the nodule biomass and leghemoglobin protein content in mungbean and the reductions were variable depending upon the *Rhizobium* strain^51^. There have also been reports of decreased nodule numbers and leghemoglobin levels in chickpea under salinity, which decreased the enzyme nitrogenase’s activity^52^. If there is no external supplementation of the nitrogen source under salinity, then with reduced leghemoglobin levels, the limited nitrogen fixing capacity results in nitrogen deficiency^34^.

The plant usable ammonium form of N can also be produced from nitrite and NR enzyme converts nitrate to nitrite^53^. Salt stress influenced the NR activity in rice. Salt-sensitive cultivars showed a reduction but it increased in salt-tolerant cultivars. Authors reported that this varied behaviour was brought on by the presence of differing numbers of GATA elements in the NR gene’s upstream region^54^. Salt environment reduced NR activity in wild as well as in cultivated soybean cultivars^55^. The data from the present work showed an increasing trend of NR with salt water application and similar pattern has been documented in chickpea^56^. In another report on chickpea a decreasing trend with salinity has been reported but the chickpea plants inoculated with *Rhizobium* culture showed an increasing trend and in the present experiment also *Rhizobium* culture was used^57^. Moreover, analysis of saline water used in the present study revealed the presence of nitrate ions, which is documented in our earlier report^15^. Thus, enhancement in the NR activity with salinity is dependent upon the presence of nitrate ions and *Rhizobium* culture.

Salt affection reduced the N content in chickpea leaves, but only sensitive genotypes showed a substantial reduction. Similarly, salt water reduced N content in all the parts (root, stem and leaves) of *Brassica* plants, but tolerant cultivars exhibited less reduction and authors associated it with RWC characteristic the more the capacity of the plant to ingest water, the more is the nitrogen absorption^58^. A report on chickpea documented that N content during salt stress is highly dependent on the genotypic nature^59^. Researchers in the past have noted that N fixation process is affected negatively under salt stress^60,61^. The reason may be degradation of leghemoglobin protein^62^a reduction in tissue defence against ROS^63^ammonium assimilation routes being inhibited, especially as a result of a decline in glutamine synthetase activity^64^etc. Thus, if an external N source is provided to the plant in such conditions, it can help the plant to maintain the optimum N levels.

The main objective of this study was to find out the association between the various nodule traits and the nitrogen present in the above ground parts. The correlation analysis revealed that a strong positive correlation of nodule number, fresh weight, dry weight, ψ_w_, ψ_s,_ RWC, membrane stability and leghemoglobin content and a strong negative correlation of H_2_O_2_, MDA and Na^+^/K^+^ ratio exits with the total N present in the above ground parts. Surprisingly, all the antioxidative enzymes (SOD, CAT, APX and POX) and NR enzyme were negatively associated with the total N content. These findings suggest that under salinity, nitrogen present in leguminous plants is primarily derived from symbiotic nitrogen fixation. NR activity increased with salinity and should provide N to plants but it was found negatively correlated with same, thus the conversion of nitrite into ammonia might be hampered by the salinity stress. Literature documents that salt stress adversely impacts nitrogen fixation in leguminous plants due to a combination of factors, including salt concentration and duration, rhizobia symbiont strain, symbiotic interactions, symbiosome activity, low nodulation, poor nodule growth and development, nitrogenase dysfunction, and leghemoglobin degradation.^65^. Basically, the N content is directly proportional to the number of healthy nodules, more is the healthy nodules more is N content^66^. The same is true with the present investigation and also it is suggested that it the symbiotic nitrogen fixation in the nodules is responsible for the N present in the plant parts not the high activity of the NR enzyme. Proteomics study on salt affected alfalfa (*Medicago sativa* L.) revealed that the plants with the active nodules represented an upfold change in cell wall remodelling and antioxidative linked proteins and downfold change in the proteins linked to protein biosynthesis and degradation and the authors suggested that symbiosis provides the capacity to host plant so that it can adjust key processes like, better efficiency to use resources and energy, oxidative balance and ion homeostasis maintenance^67^.

## Conclusion

In our investigations, we found that saline irrigations impose stress on nodules, disrupting their physiological functions and impacting nitrogen-fixing capacity. The maintenance of an optimal Na^+^/K^+^ ratio and water relations, alongside water and osmotic potentials, emerge as crucial traits for ensuring nodule health. Furthermore, our observations indicate that symbiotic nitrogen fixation within nodules contributes significantly to nitrogen content in plant parts, rather than the heightened activity of the NR enzyme. Consequently, the presence of healthier nodules correlates with increased nitrogen fixation, enhancing its availability to the plant— representing a valuable trait for breeding salt-tolerant legumes.

## Supplementary Information

Below is the link to the electronic supplementary material.

Supplementary Material 1

## Data Availability

All data supporting the findings of this study are available within the paper and its Supplementary Information. qPCR results file is provided as “Supplementary file - qPCR results”.
